# Efficacy of probiotics in the treatment of allergic diseases: a meta-analysis

**DOI:** 10.3389/fnut.2025.1502390

**Published:** 2025-03-04

**Authors:** Zhang Xi, Xing Fenglin, Zhao Yun, Li Chunrong

**Affiliations:** ^1^Chengdu Women’s and Children’s Central Hospital, School of Medicine, University of Electronic Science and Technology of China, Chengdu, China; ^2^Chengdu Qingyang District Maternal and Child Health Hospital, Chengdu, China; ^3^Chengdu Children’s Specialized Hospital, Chengdu, China

**Keywords:** probiotics, allergic diseases, asthma, rhinitis, eczema

## Abstract

**Background information:**

Allergic diseases are an increasingly serious health issue worldwide, affecting not only the physiological health of patients but also significantly reducing their quality of life, thereby imposing a substantial economic burden on families and society. According to data from the World Health Organization, the incidence of allergic diseases has risen markedly over the past few decades, particularly among children and adolescents, making it a significant public health challenge. Although several clinical studies have explored the effects of probiotics in the treatment of food-induced allergies and allergic diseases, the results have been inconsistent. Some studies indicate positive effects, while others fail to demonstrate their efficacy. Therefore, a systematic evaluation of the effectiveness of probiotics in allergic diseases is particularly important. Some studies indicate that patients with food allergies may also experience respiratory symptoms, and certain foods may be associated with the onset or exacerbation of allergic rhinitis and asthma. Diseases such as allergic rhinitis, asthma, and atopic dermatitis are characterized by inappropriate immune responses to typically harmless environmental allergens. The incidence of these diseases is continuously rising in urban populations, prompting researchers to extensively explore novel therapeutic strategies that can effectively modulate immune responses.

**Objective:**

The aim of this study is to systematically assess the effectiveness of probiotics in the treatment of allergic diseases. By integrating the results of existing clinical studies, we hope to provide a clearer scientific basis for the treatment of allergic diseases.

**Methods:**

We conducted a comprehensive literature search in databases such as PubMed for articles published before the end of 2023 that evaluated the effectiveness of probiotics in treating allergic diseases. Inclusion criteria focused on studies reporting binary outcomes related to the efficacy of probiotics in patients with food allergies, asthma, allergic rhinitis, or atopic dermatitis. Data were collected using Excel software, and the Review Manager software was used to analyze the collected binary variable data. The effectiveness of probiotics was assessed by calculating the risk ratio (RR) and its 95% confidence interval (CI). Heterogeneity among studies was evaluated using the I^2^ statistic, and publication bias was assessed through funnel plots.

**Results:**

The analysis of the aggregated binary data indicates that probiotics significantly improve clinical outcomes in patients with allergic diseases. Additional analysis using continuous variables (scores) further demonstrates the effectiveness of probiotics in alleviating allergic diseases. Subgroup analyses show that probiotics are effective in treating various common conditions, with two specific probiotics strains being particularly effective for allergic diseases.

**Conclusion:**

We included literature involving pediatric patients with common allergic diseases, Probiotics can help treat allergic diseases by modulating immune mechanisms, but allergic diseases are typically caused by multiple factors and individual variations, however, allergic diseases are typically caused by multiple factors and individual variations, so they should not be used as the sole treatment method.This meta-analysis provides evidence supporting the effectiveness of probiotics in various allergic diseases. The findings suggest that probiotics can serve as a beneficial adjunctive therapy for the treatment of these conditions.

**Systematic review registration:**

https://clinicaltrials.gov/, CRD42024586317.

## 1 Introduction

Allergic diseases have become a significant global public health issue, affecting the health of millions of people and leading to substantial morbidity and healthcare costs. Food allergies and respiratory allergies are common conditions, and their prevalence is on the rise. Some studies indicate that patients with food allergies may also experience respiratory symptoms, and certain foods may be associated with the onset or exacerbation of allergic rhinitis and asthma (1). Diseases such as allergic rhinitis, asthma, and atopic dermatitis are characterized by inappropriate immune responses to typically harmless environmental allergens. The incidence of these diseases is continuously rising in urban populations, prompting researchers to extensively explore novel therapeutic strategies that can effectively modulate immune responses (2–5).

Despite a growing body of literature supporting the use of probiotics in managing allergic diseases, the results of individual studies have been inconsistent. Some clinical trials report significant improvements in symptoms and quality of life (6–8), while others demonstrate only weak effects (9). This variability may be attributed to differences in study design, probiotic strains, dosages, and patient populations. Therefore, there is an urgent need for a comprehensive evaluation of the existing evidence to clarify the efficacy of probiotics in the treatment of allergic diseases (10, 11).

This meta-analysis aims to systematically review and synthesize existing randomized controlled trials (RCTs) and clinical studies on the effects of probiotics on allergic diseases. It seeks to provide a clearer understanding of the therapeutic potential of probiotics, identify factors that may influence treatment outcomes, and offer insights for future research directions in this evolving field (12).

## 2 Materials and methods

### 2.1 Search strategy and selection criteria

This study follows the “Preferred Reporting Items for Systematic Reviews and Meta-Analyses (PRISMA)” guidelines to ensure that the design and reporting of our meta-analysis meet international standards (13). Given that this study involves secondary data analysis, ethical committee approval is not required. Literature published in 2023 and earlier was retrieved from the PubMed. We used search terms including: allergic diseases or asthma or rhinitis, probiotics, and eczema. We included literature from RCTs and other relevant studies that provided full-text articles, while excluding studies that contained only abstracts, unpublished data, or were in languages other than English.

We included literature involving pediatric patients with common allergic diseases, specifically studies reporting on diagnosed children. This analysis included research on oral probiotics, comparing this intervention with a placebo control group to assess whether allergic manifestations relieved post-intervention. We explored whether different diseases influenced the control of allergic conditions following probiotic use and evaluated the impact of single versus combined probiotic strains on health outcomes. Two personnel independently screened the literature based on the inclusion and exclusion criteria. In case of disagreements between the two reviewers, a third reviewer was consulted to mediate and reach a consensus.

## 3 Data extraction

The following items were extracted from the included literature: ① basic study information, including the first author and year of publication; ② study design; ③ number of cases; ④ type of probiotics. References from the initially retrieved literature that met the study criteria were also subjected to data extraction (14, 15). Two personnel independently extracted relevant data from the included studies and verified the accuracy of the data entry. In case of discrepancies, the researchers consulted with each other to reach a consensus. When a study reported multiple groups within a single trial, only the relevant groups were included.

## 4 Quality assessment

The Cochrane tool for assessing the risk of bias in randomized trials was used, with two personnel independently evaluating the quality of the literature across six bias domains (16): ① selection bias: random sequence generation and allocation concealment; ② performance bias: blinding of participants and personnel; ③ detection bias: blinding of outcome assessment; ④ attrition bias: incomplete outcome data; ⑤ reporting bias: selective reporting; ⑥ other bias. The risk of bias for each domain is categorized as low, high, or unclear. A study is considered to have a low risk of bias if each domain is clearly described. If at least one domain is assessed as high risk, the study is deemed to have a high risk of bias. If at least one domain’s risk is unclear, the study is classified as having unclear risk. Discrepancies will be resolved by consulting a third reviewer.

## 5 Statistical analysis

**Figure 1:**
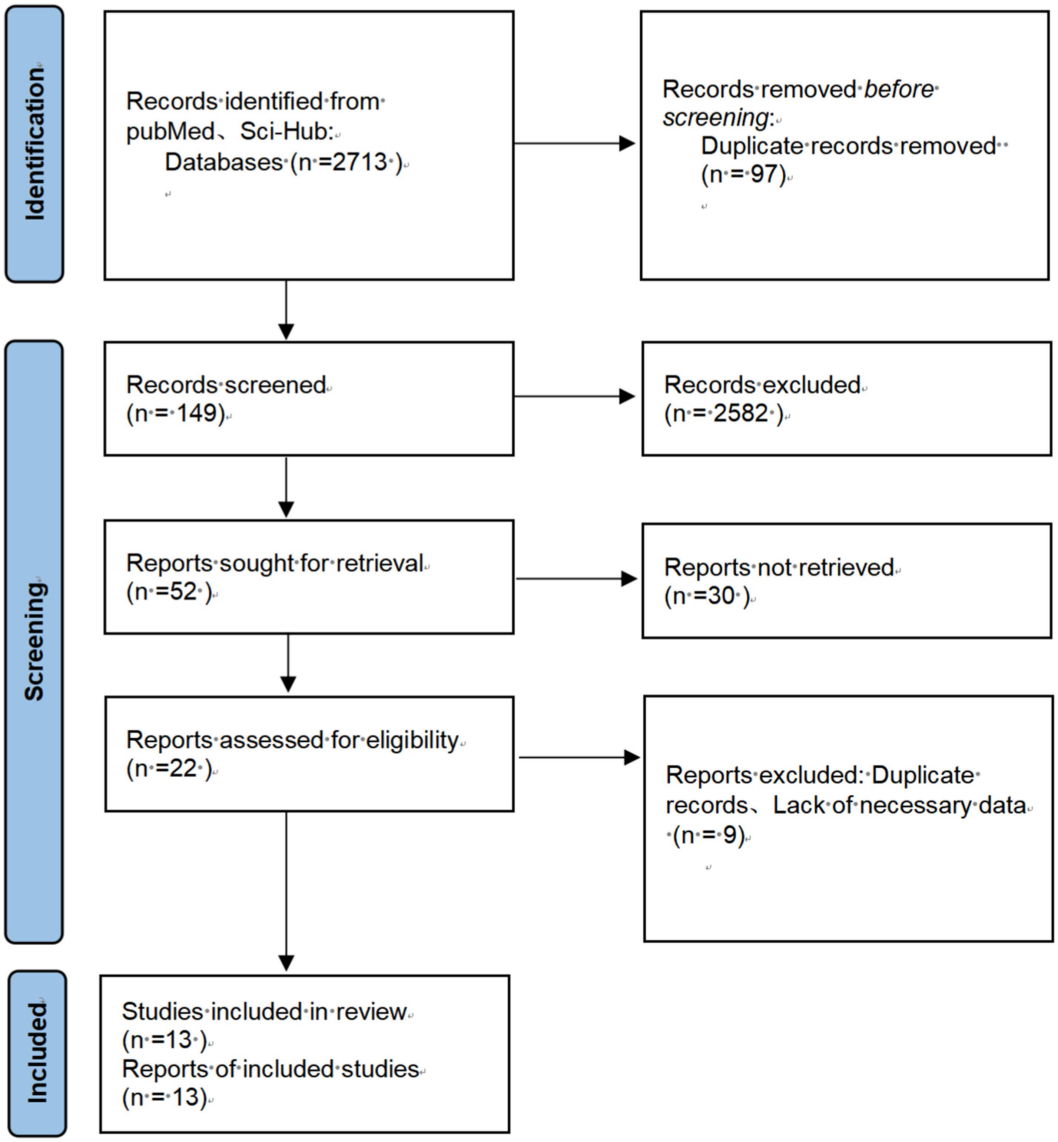
Flowchart of the search process for articles included.

Data were collected using Excel software and analyzed using Review Manager Software. The effectiveness of probiotics was assessed by calculating the risk ratio (RR) and its 95% confidence interval (CI). A forest plot was used to evaluate heterogeneity between studies. A fixed-effect model was applied for studies with low heterogeneity (*I*^2^ < 50%, *p* > 0.1), whereas a random-effects model was used for studies with high heterogeneity (*I*^2^ ≥ 50%, *p* ≤ 0.1). A *p*-value of <0.05 was considered statistically significant. A funnel plot was employed to explore potential publication bias. Heterogeneity between studies was assessed using the *I*^2^ statistic, and publication bias was evaluated through the funnel plot (Figure 1).

## 6 Results

### 6.1 Characteristics of patients

A total of 2,731 relevant studies were initially retrieved from the database. After removing 97 duplicate studies, 2,582 articles were excluded based on a review of titles, abstracts, and keywords. Following a full-text review, an additional 39 articles were excluded. Ultimately, 13 studies were included in the analysis (17–29).

The articles that met the inclusion criteria were incorporated into the meta-analysis, and their characteristics are presented in Table 1.

**Table 1:** Characteristics of 13 RCTs included in the meta-analysis.

Author/year	Sample	Study design	Type of probiotics	Type
Jonatas (2019)	30	quasi-experimental	*Lactobacillus reuteri*	Asthma
Yue-Sheng Chen (2010a)	105	RCT	*Lactobacillus gasseri* A5	Asthma
Yue-Sheng Chen (2010b)	94	RCT	*Lactobacillus gasseri* A5	Rhinitis
Xiao-Dong (2021)	206	RCT	*Lactobacillus*, *Bifdobacterium animalis*	Eczema
Chian-Feng (2018a)	147	RCT	*Lactobacillus paracasei*	Asthma
Chian-Feng (2018b)	147	RCT	*Lactobacillus fermentum*	Asthma
Michele (2017a)	40	RCT	Bifidobacteria mixture	Rhinitis
Michele (2017b)	40	RCT	Bifidobacteria mixture	Rhinitis
Miisa Komulainen (2023a)	195	RCT	Lacticaseibacillus rhamnosus, *Bifidobacterium animalis*	Asthma
Miisa Komulainen (2023b)	195	RCT	Lacticaseibacillus rhamnosus, *Bifidobacterium animalis*	Eczema
Rikke (2019a)	260	RCT	*Lactobacillus rhamnosus*, *Bifidobacterium animalis*	Asthma
Rikke (2019b)	241	RCT	*Lactobacillus rhamnosus*, *Bifidobacterium animalis*	Eczema
Rikke (2019c)	260	RCT	*Lactobacillus rhamnosus*, *Bifidobacterium animalis*	Rhinitis
Gareth Davies (2018a)	370	RCT	Lactobacilli, Bifdobacteria	Asthma
Gareth Davies (2018b)	370	RCT	Lactobacilli, Bifdobacteria	Eczema
Erica (2020)	255	RCT	Bifidobacterium, *Streptococcus thermophilus*	Eczema
Niers (2009)	102	RCT	Bifidobacterium, *Lactococcus lactis*	Eczema
Kaarina (2007)	925	RCT	*Lactobacillus rhamnosus*, Bifidobacterium, Propionibacterium freudenreichiis	Eczema
Bozena (2021)	96	RCT	*Lactobacillus rhamnosus*, *Lactobacillus casei*	Eczema
Lorenzo (2022)	422	RCT	Ligilactobacillus salivarius, *Bifidobacterium breve*	Asthma

### 6.2 Quality assessment of the literature

**Figure 2:**
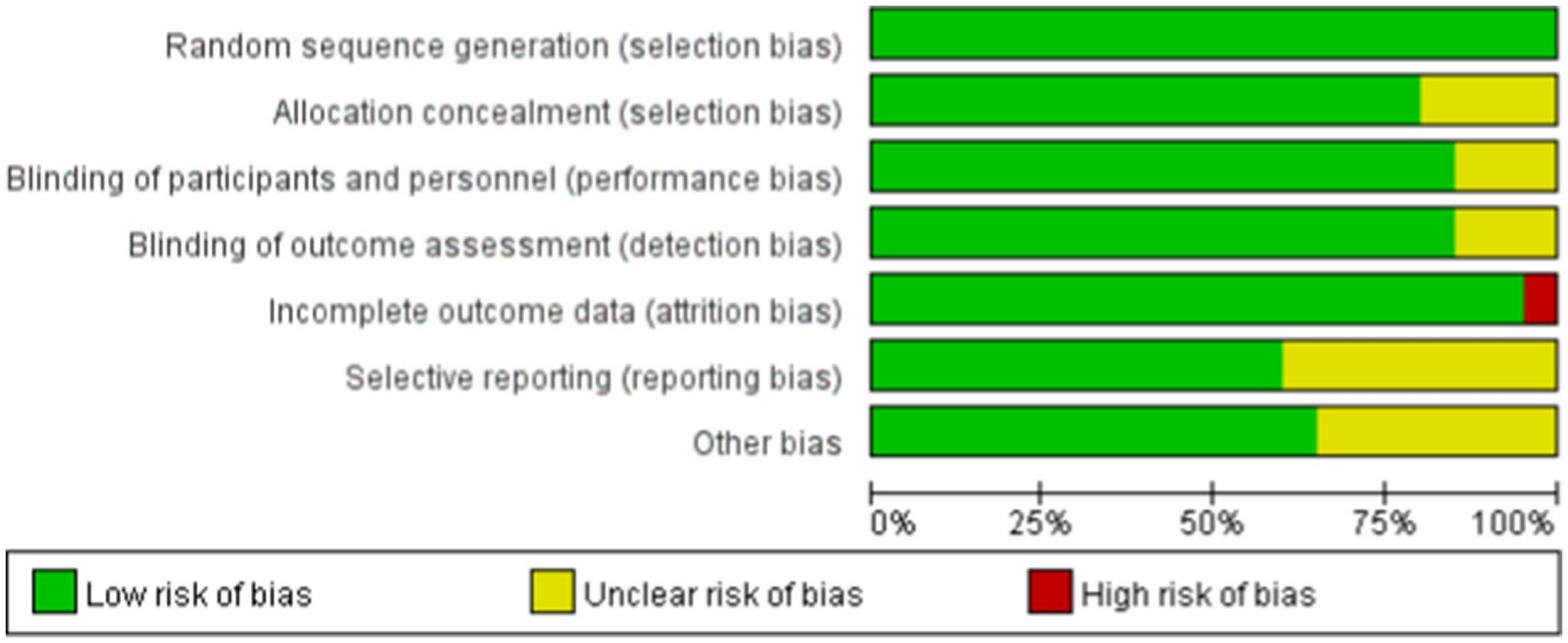
Risk of bias summary. The quality assessment of each literature has been shown. The color green, yellow and red mean low, high and unclear risk of bias, respectively, (Color online).

**Figure 3:**
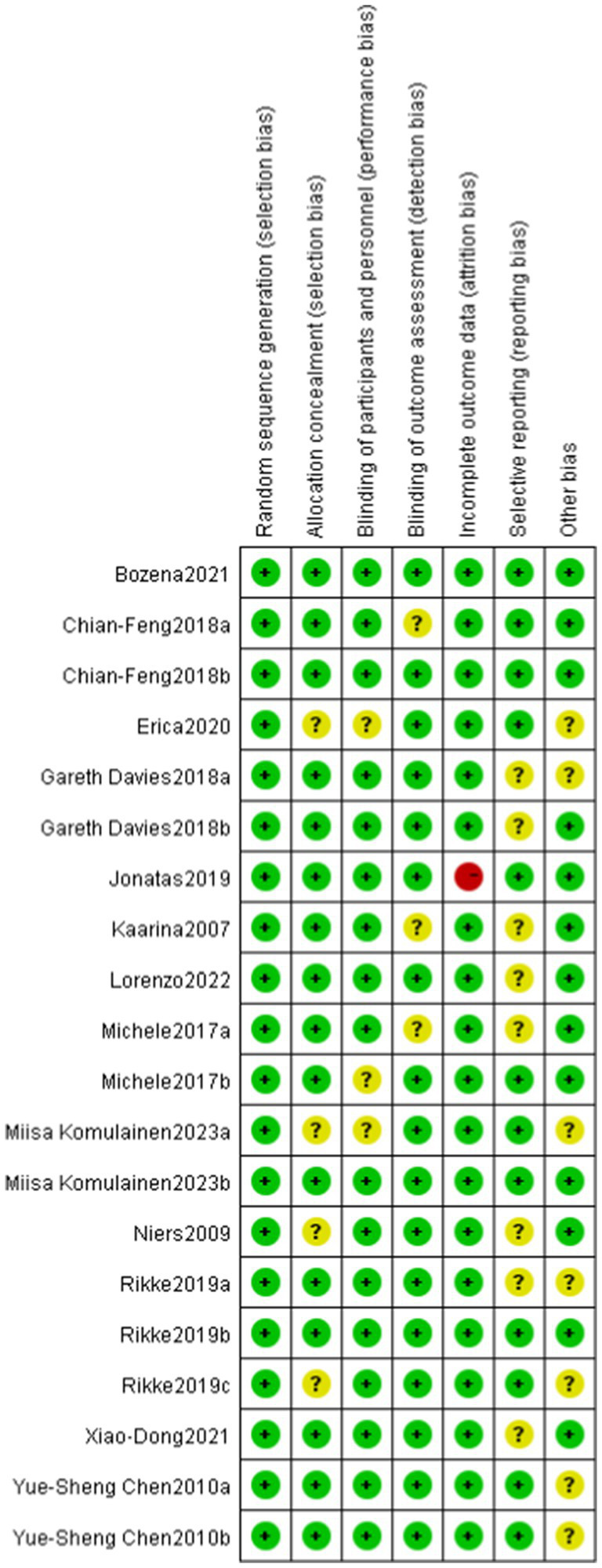
Risk of bias assessment for included studies. Low risk of bias (+), high risk of bias (−), unclear risk of bias (?).

Among the 13 RCTs, 12 were randomized double-blind trials, and 1 was a quasi-experimental study. The risk of bias for these studies was assessed using the Cochrane tool, as illustrated in Figures 2, 3.

### 6.3 Effects of probiotics on allergic diseases

This study evaluated the impact of probiotics on the risk of developing allergic diseases. The overall results indicated that the probiotics group had a 25% lower risk of allergic diseases compared to the control group (RR = 0.75), with this result being statistically significant (*p* = 0.009). The heterogeneity analysis revealed a moderate level of heterogeneity among studies, with an I^2^ statistic of 51%. Despite this, the relative risks in the most studies were less than 1, reinforcing the conclusion that the incidence of allergic diseases was significantly lower in the probiotics group compared to the control group.

**Figure 4:**
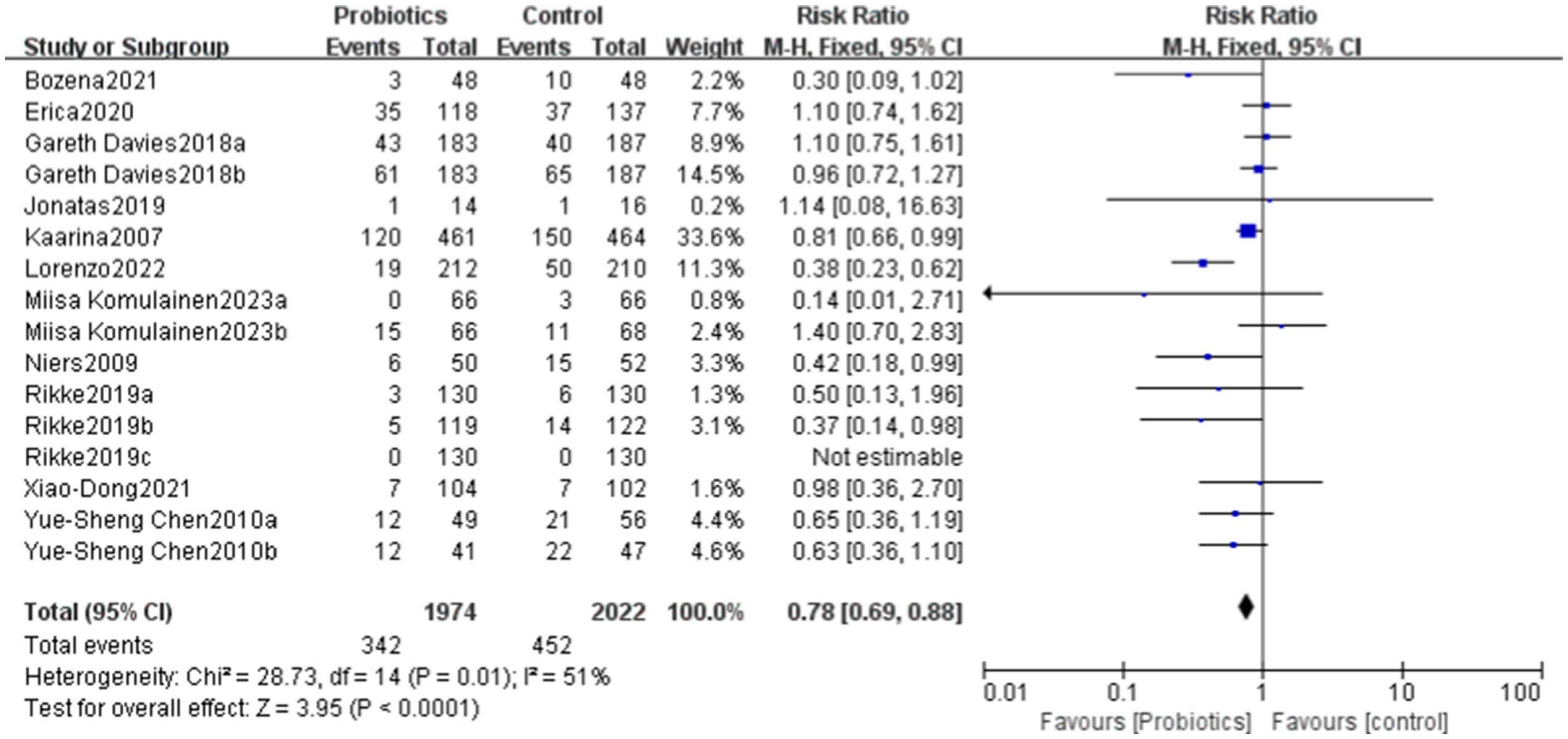
Forest plot showing the effectiveness of probiotics in treating allergic diseases.

**Figure 5:**
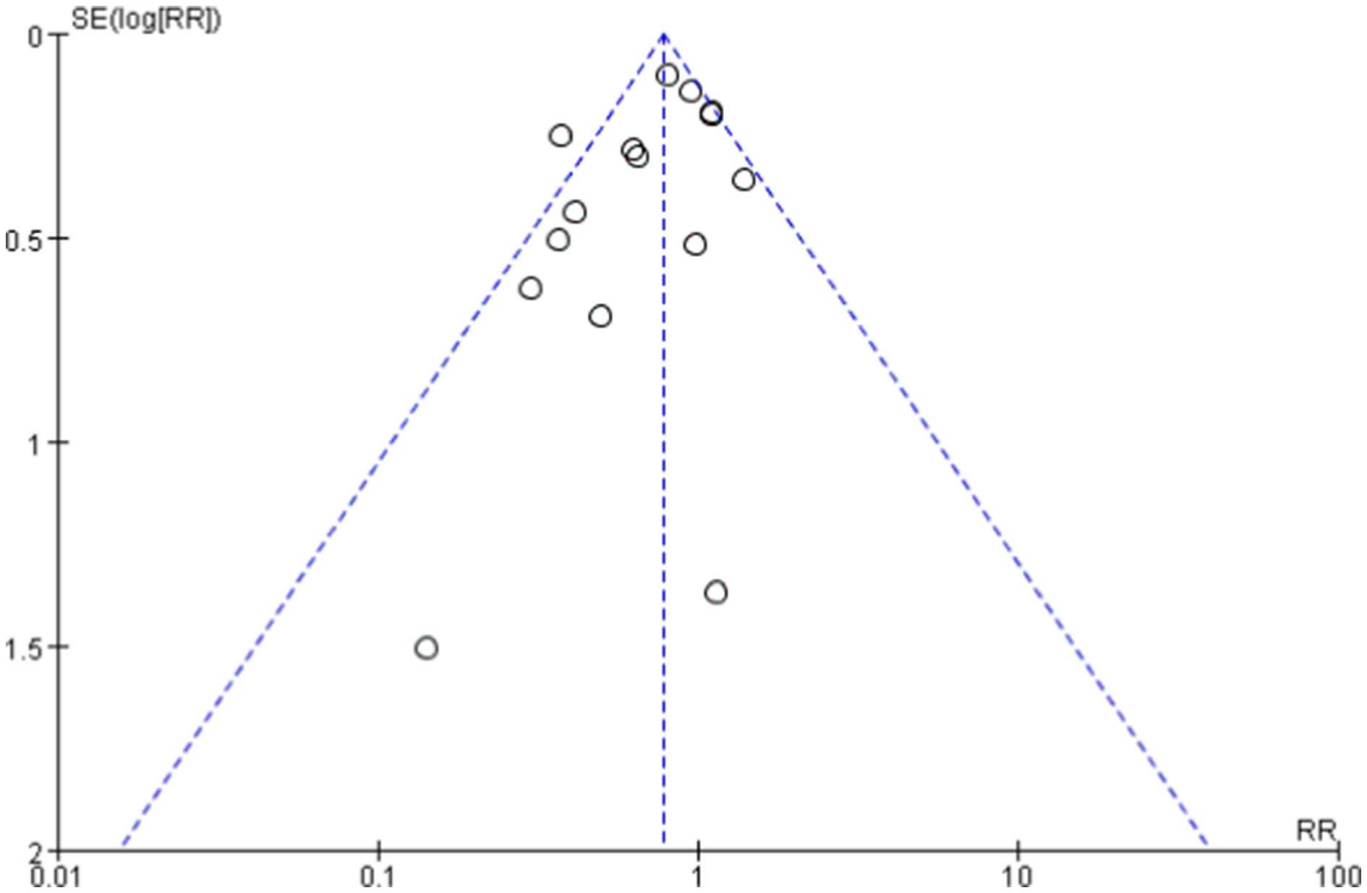
Funnel plot showing publication bias.

The overall effect Z-value was 2.63 (*p* = 0.009), indicating a significant association between the probiotics use and a reduced risk of allergic diseases. Additionally, the funnel plot analysis showed good overall symmetry, suggesting a low likelihood of publication bias, and enhancing the reliability of the study results (Figures 4, 5).

### 6.4 Comparison of differences in various scoring systems between probiotics and control groups

**Figure 6:**
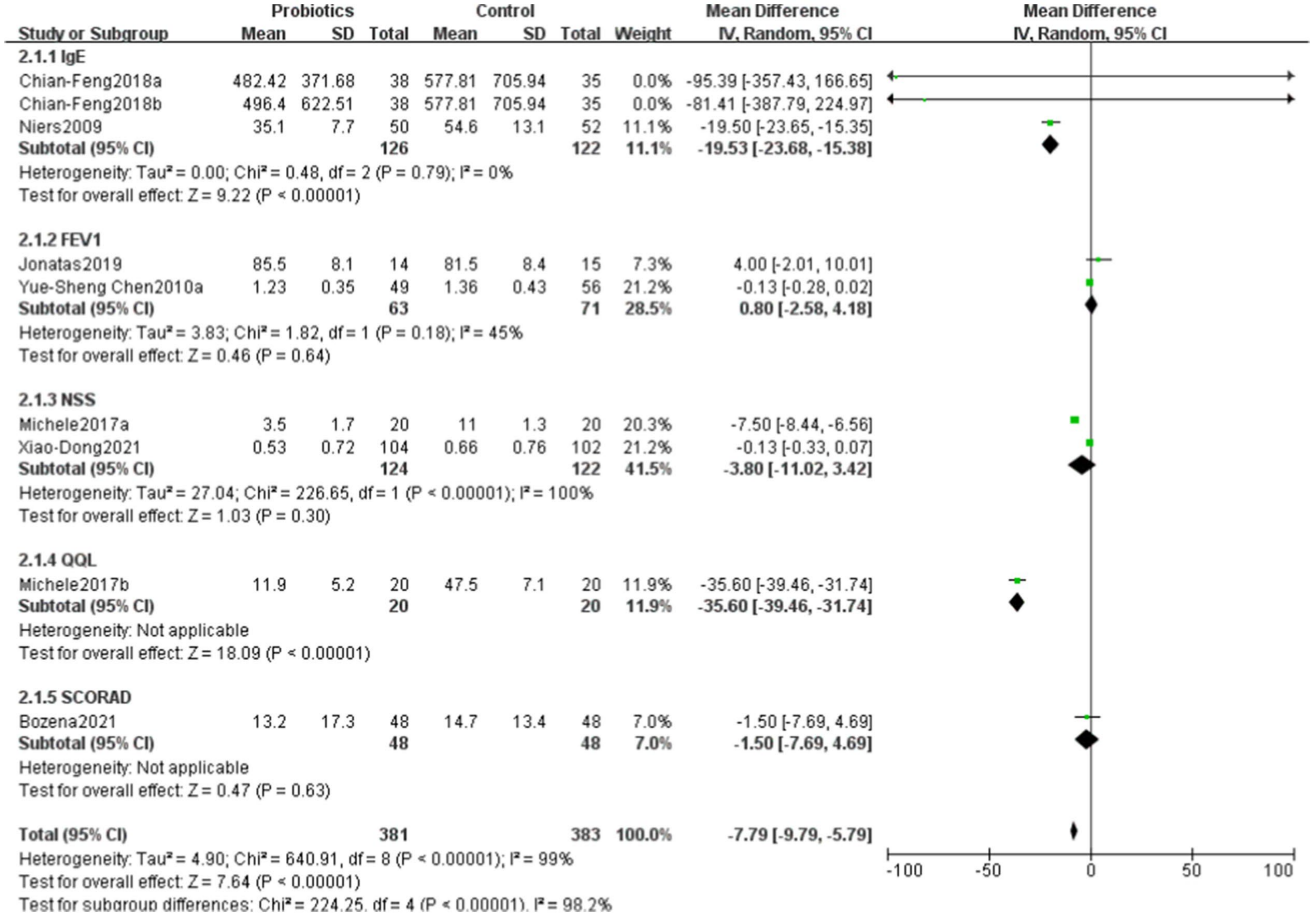
Changes in scoring system following probiotics use for allergic diseases.

**Figure 7:**
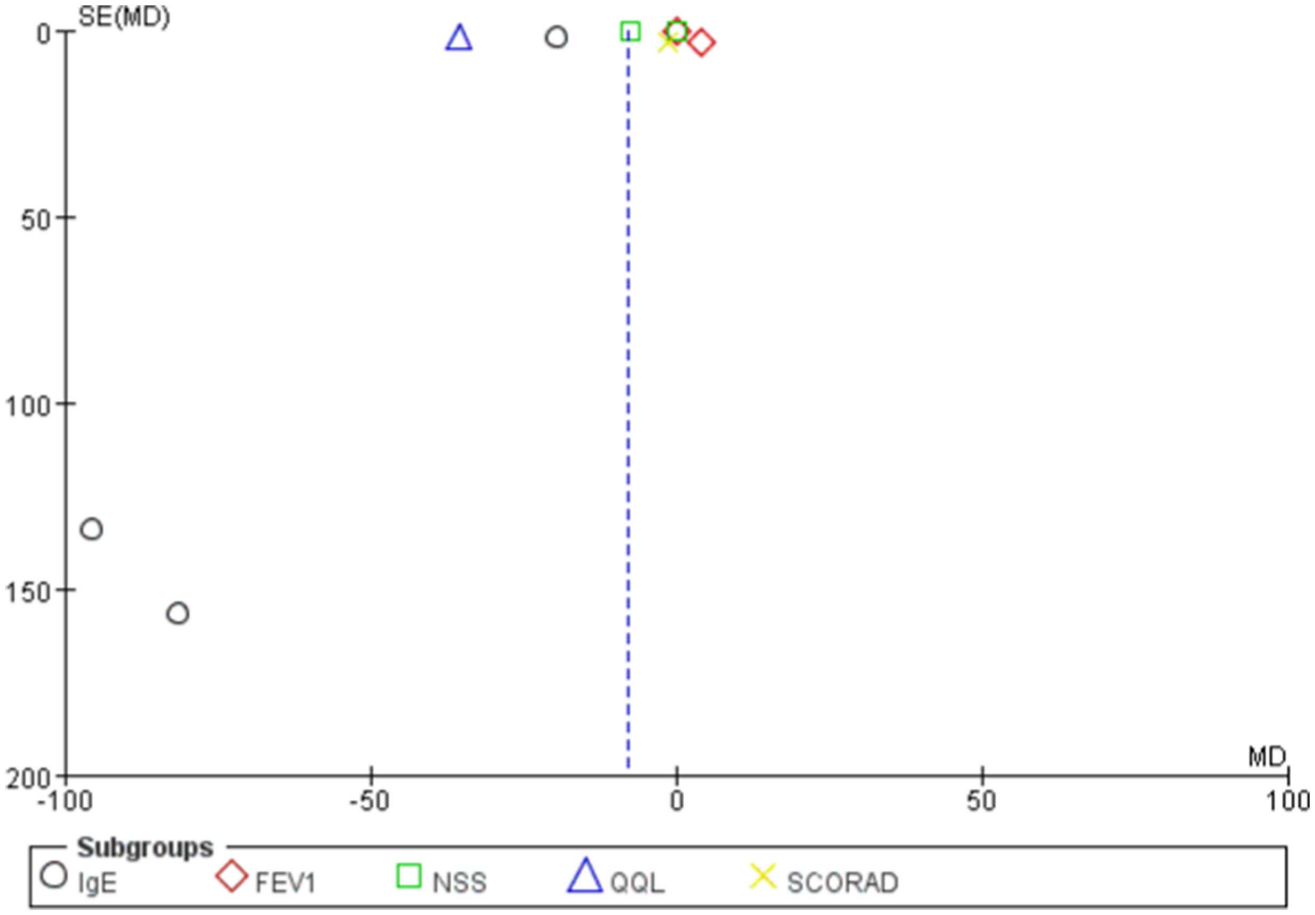
Funnel plots in scoring system.

The overall mean difference was-7.79 (95% CI: −9.79, −5.79), indicating that the symptom scores in the probiotics group were significantly lower than those in the control group, with statistical significance (*p* < 0.00001). The overall effect test showed a Z-value of 7.64 (*p* < 0.00001), indicating that the overall effect was statistically significant. Overall, the funnel plot did not demonstrate any obvious publication bias, and probiotics significantly reduced symptom scores (Figures 6, 7).

### 6.5 Analysis of the use of one, two, and three probiotic strains in the treatment of allergic diseases

In the studies using one probiotic strain, a total of 25 events in the probiotics group and 44 events in the control group were included. The RR was 0.65, with a CI of [0.43, 0.98]. The overall effect in this group was significant (*p* = 0.04), indicating that the risk in the group using one probiotic strain was significantly lower than that in the control group.

In the studies using two probiotics strains, a total of 162 events in the probiotics group and 221 events in the control group were included. The RR was 0.74, with a CI of [0.62, 0.89]. The overall effect in this group was also significant (*p* = 0.001), indicating that the risk in the group using two probiotics strains was significantly lower than that in the control group.

In the studies using three probiotics strains, the total number of events was 120 in the probiotics group and 150 in the control group. The RR was 0.81, with a CI of [0.66, 0.99]. The overall effect in this group was similarly significant (*p* = 0.04), indicating that the risk in the group using three probiotics strains was significantly lower than that in the control group.

**Figure 8:**
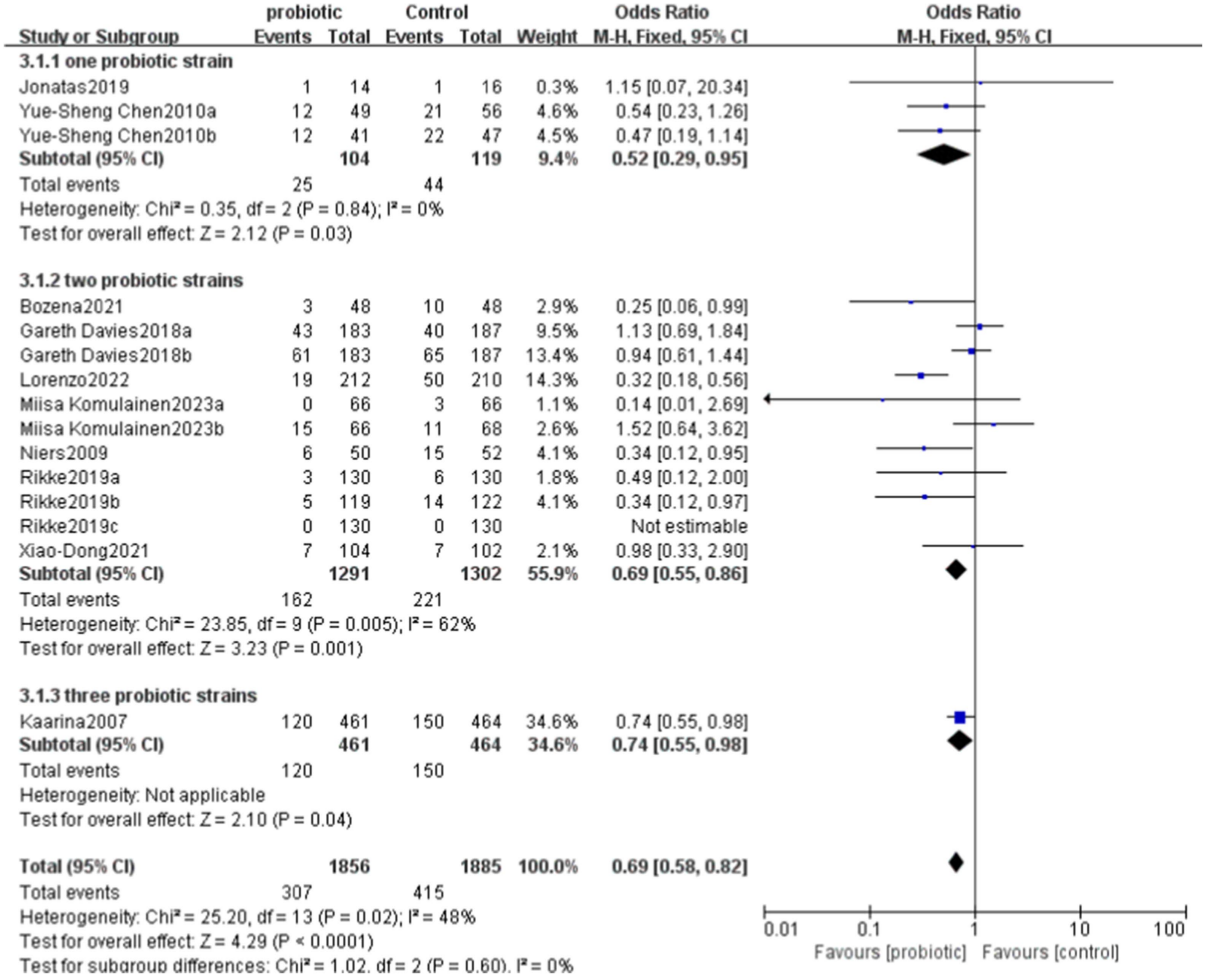
Forest of evaluated the effects of single probiotics versus combined probiotics outcomes.

**Figure 9:**
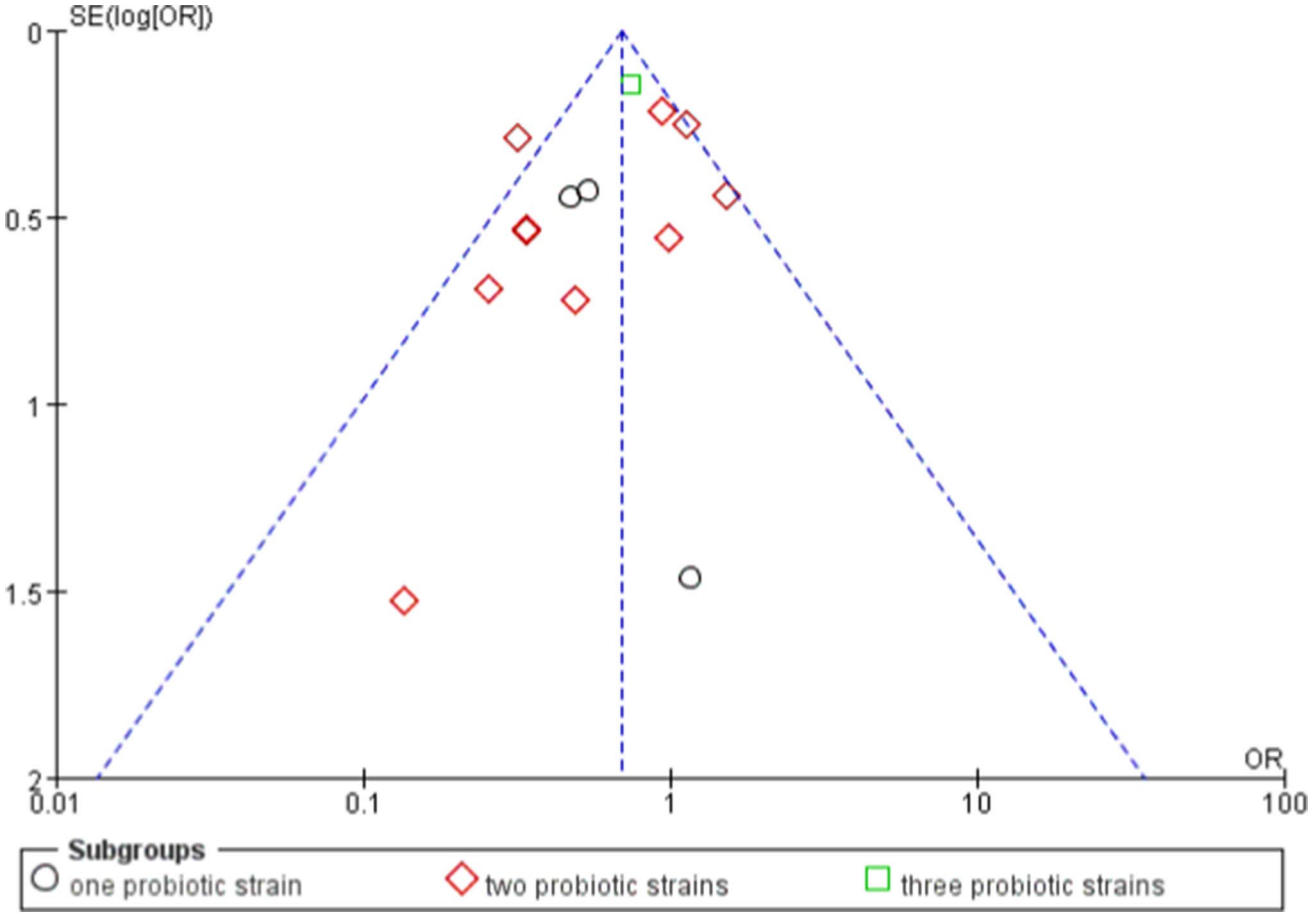
Funnel of evaluated the effects of single probiotics versus combined probiotics outcomes.

Combining the results of all studies, the RR was 0.76, with a CI of [0.67, 0.86], *p* < 0.0001, demonstrating that probiotics significantly reduced the risk of a certain outcome (Figures 8, 9).

The probiotic treatment strategies collected in this study are mainly categorized into four types: those based on lactobacilli, those based on bifidobacteria, the combination of lactobacilli and bifidobacteria, and the combined treatment of lactobacilli, bifidobacteria, and propionibacteria. Lactobacilli exhibit antibacterial properties, regulate gut microbiota, and enhance immune function, while bifidobacteria primarily contribute to maintaining intestinal barrier integrity, inhibiting pathogenic bacteria, and alleviating gut inflammation. The combination of these two types of probiotics can produce synergistic effects. Propionibacteria, on the other hand, exert additional anti-inflammatory and immunomodulatory effects through their metabolic byproducts, such as propionic acid and acetic acid. However, these mixed treatment strategies may face challenges such as metabolic competition between strains, differences in environmental sensitivity among species, and formulation stability issues. Furthermore, variations in the gut microbiota composition of different individuals may also impact the efficacy of mixed probiotic treatments.

### 6.6 The impact of probiotics on different diseases

**Figure 10:**
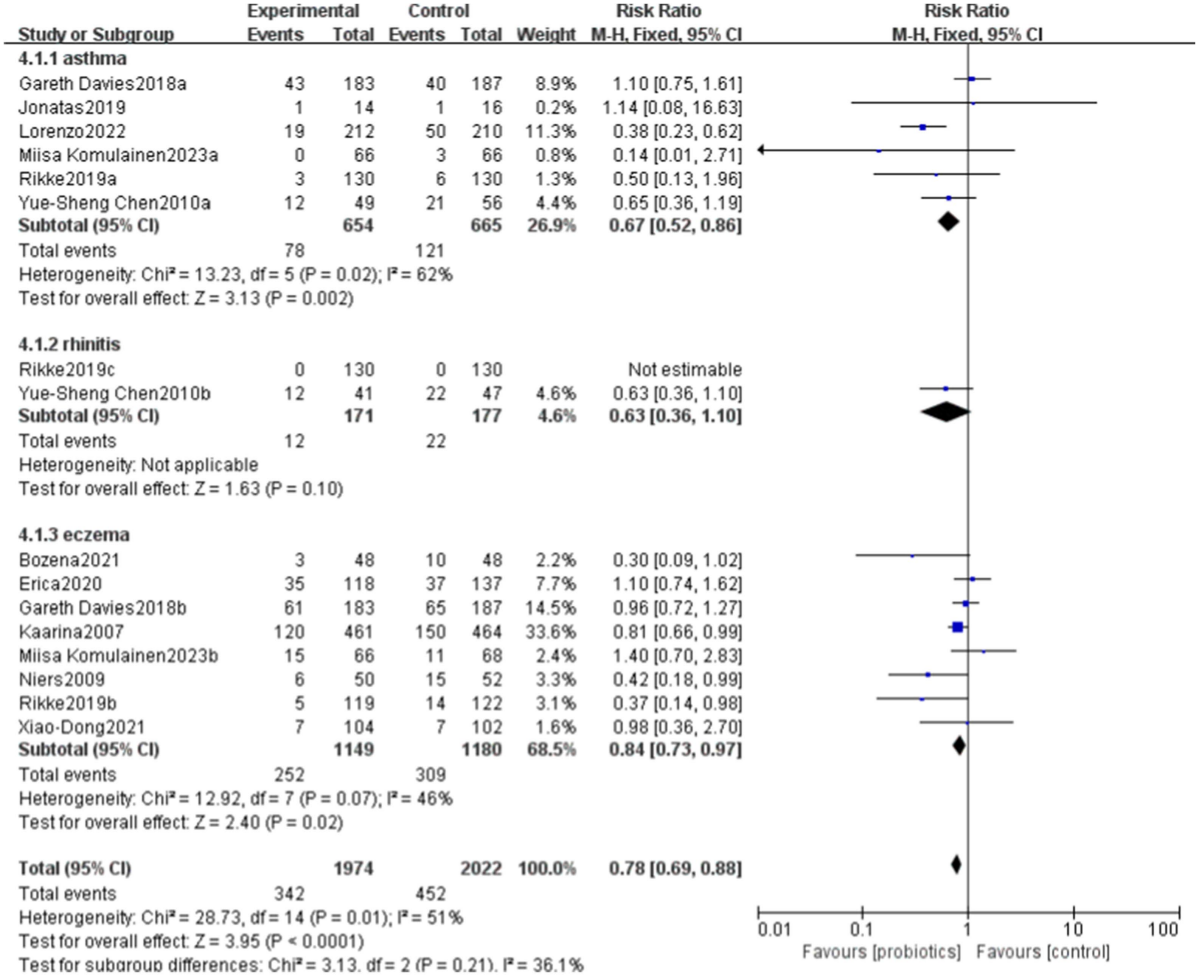
Forest plot of the effects of probiotics on different diseases.

**Figure 11:**
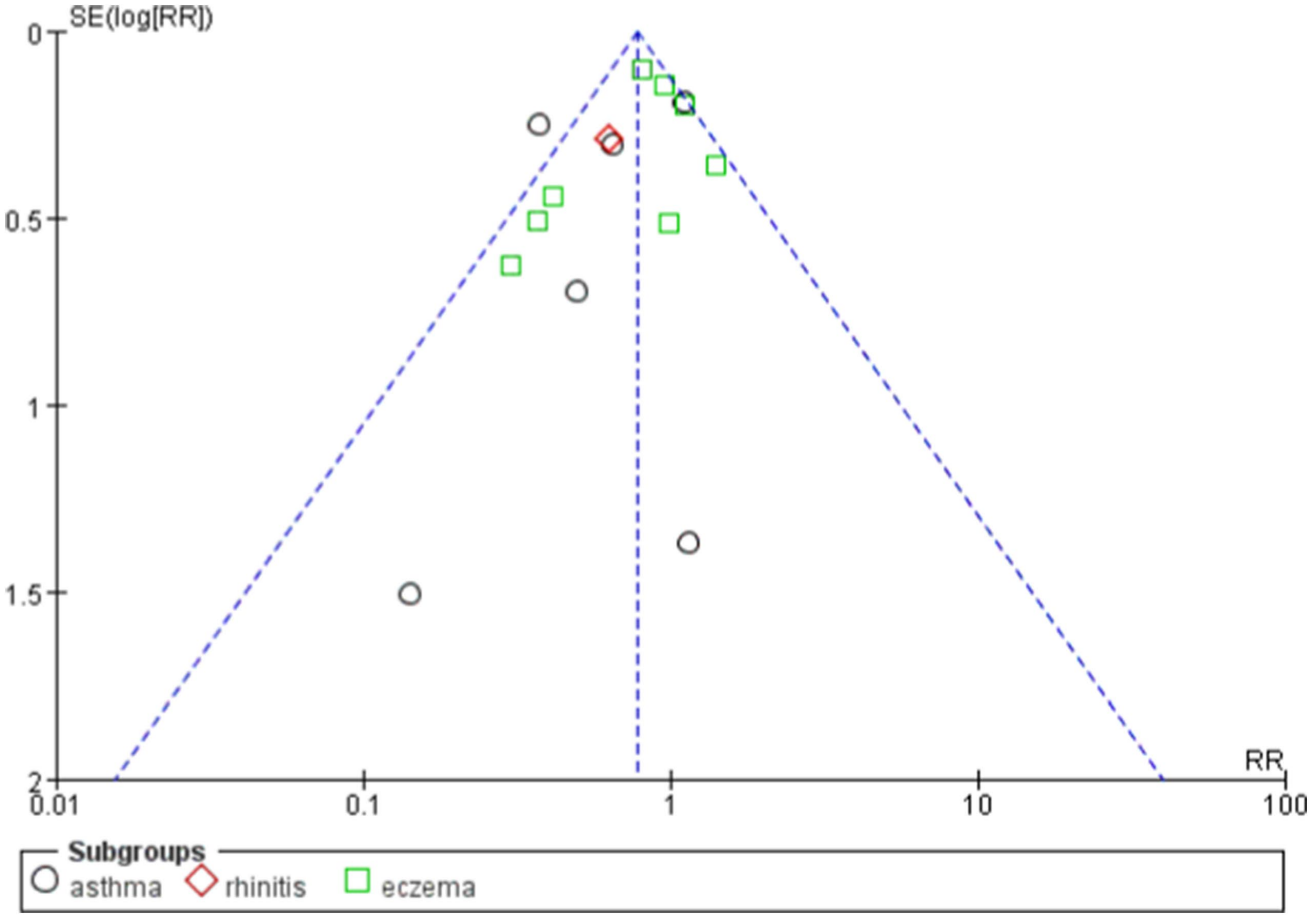
funnel plot of the effects of probiotics on different diseases.

Probiotics have demonstrated benefits in reducing allergic diseases, particularly asthma and eczema. Overall, the funnel plot shows a certain degree of symmetry (Figures 10, 11).

## 7 Discussion

We included literature involving pediatric patients with common allergic diseases, Probiotics can help treat allergic diseases by modulating immune mechanisms, but allergic diseases are typically caused by multiple factors and individual variations (30), so they should not be used as the sole treatment method (31). This study assessed the effects of probiotics on different scoring systems through a meta-analysis. The results showed that the symptom scores in the probiotics group were significantly lower than those in the control group, indicating a significant negative correlation between probiotics use and symptom improvement. This finding is consistent with existing literature (32, 33), supporting the potential role of probiotics in improving allergic diseases and other related symptoms.

In various scoring systems, the results for IgE and quality of life were particularly notable, indicating that probiotics may reduce allergic symptoms and improve quality of life by modulating immune responses and enhancing the gut microbiome (34). The mechanisms of action of probiotics (35) may be related to their ability to promote intestinal barrier function, suppress inflammatory responses, and regulate the host immune system. Studies have shown that certain probiotic strains can lower IgE levels, thereby alleviating allergic symptoms. However, the results for FEV1, NSS, and SCORAD were not statistically significant, which may reflect the complexity and diversity of these scoring systems. FEV1, as an indicator of lung function, may be influenced by various factors, including environmental factors, individual differences, and the severity of underlying diseases. Therefore, the effects of probiotics on improving lung function require further investigation.

Although the results of this study indicate that probiotics are effective in improving common allergic diseases, the clinical significance of these findings suggests that probiotics may serve as an adjunctive treatment. However, further research targeting different populations, symptoms, and probiotic strains is still necessary to clarify their optimal application scenarios and mechanisms. Additionally, it is noteworthy that some studies have shown less than ideal results following probiotic interventions; for example, certain clinical trials failed to demonstrate the effectiveness of probiotics on specific allergic symptoms, which may be related to individual differences, the types of probiotics, and their dosages. These negative results further emphasize the need for cautious evaluation of the role of probiotics in clinical applications (36, 37).

Some studies indicate that patients with food allergies may also experience respiratory symptoms, and certain foods may be associated with the onset or exacerbation of allergic rhinitis and asthma (1). Diseases such as allergic rhinitis, asthma, and atopic dermatitis are characterized by inappropriate immune responses to typically harmless environmental allergens.

Eczema and asthma, as common allergic diseases, exhibit certain similarities in their pathological mechanisms; however, the effects of probiotics on these two conditions show significant differences. Current studies and meta-analyses provide some evidence supporting the beneficial effects of probiotics on eczema (38). Certain strains may help reduce the incidence of eczema or alleviate its symptoms by modulating the gut microbiota, enhancing immune tolerance, and reducing the levels of inflammatory cytokines. However, the results of this study suggest that probiotics may contribute to the improvement of asthma. Some studies have also shown positive effects of probiotics on asthma (39, 40), while others have reported limited or no significant benefits (41). The effectiveness of probiotics in asthma management may be influenced by various factors, including the specific conditions of the patients, the type and dosage of probiotics used, as well as the duration of the treatment.

In summary, this meta-analysis provides strong evidence for the effectiveness of probiotics in the treatment of allergic diseases. However, the heterogeneity among studies and the non-significant results for certain symptom scores suggest that more detailed and systematic exploration is needed in clinical applications and future research.

## 8 Conclusion

We included literature involving pediatric patients with common allergic diseases, Probiotics can help treat allergic diseases by modulating immune mechanisms (42, 43), but allergic diseases are typically caused by multiple factors and individual variations, however, allergic diseases are typically caused by multiple factors and individual variations, so they should not be used as the sole treatment method. This meta-analysis provides evidence supporting the effectiveness of probiotics in various allergic diseases. The findings suggest that probiotics can serve as a beneficial adjunctive therapy for the treatment of these conditions.

Some studies indicate that patients with food allergies may also experience respiratory symptoms, and certain foods may be associated with the onset or exacerbation of allergic rhinitis and asthma (1). Diseases such as allergic rhinitis, asthma, and atopic dermatitis are characterized by inappropriate immune responses to typically harmless environmental allergens. This study explored the effectiveness of probiotics in reducing the risk of allergic diseases through systematic literature screening and meta-analysis. The results indicate that the use of probiotics significantly reduces the risk of allergic diseases, with a risk ratio of 0.75 (*p* = 0.009), suggesting that probiotics may play an important role in the prevention and management of allergic diseases. Although there is a certain degree of heterogeneity among the studies (*I*^2^ = 51%), the majority of the results consistently support the positive effects of probiotics.

Furthermore, the comparison of symptom scores further validated the efficacy of probiotics, showing significant improvement. These findings provide strong support for the clinical application of probiotics in allergic diseases. However, it is important to note that the heterogeneity present in the studies and the differences in scoring systems highlight the complexity and diversity of future research. Therefore, it is recommended that future studies focus on exploring different probiotic strains, dosages, and their mechanisms of action to better understand the potential of probiotics in the management of allergic diseases.

Although this analysis provides a more comprehensive evaluation, some questions remain unanswered, such as the optimal combination of different probiotic strains and dosages.

In conclusion, the results of this study provide scientific evidence for the efficacy of probiotics as an adjunctive treatment for allergic diseases, emphasizing their potential for clinical practice.

## Data Availability

The original contributions presented in the study are included in the article/supplementary material, further inquiries can be directed to the corresponding author/s.
